# Risk Factors for Rubella Transmission in Kuyu District, Ethiopia, 2018: A Case-Control Study

**DOI:** 10.1155/2019/4719636

**Published:** 2019-09-16

**Authors:** Abdulbari Abdulkadir, Tsegaye Tewelde Gebrehiwot

**Affiliations:** ^1^Public Health Emergency Management Core Unit, Jimma Zone Health Office, Oromia Region, Jimma, Ethiopia; ^2^Assistant Professor of Epidemiology, Jimma University, College of Public Health and Medical Science, Jimma, Ethiopia

## Abstract

**Background:**

Rubella is a vaccine-preventable disease associated with a significant morbidity and adverse pregnancy outcomes, mainly if acquired in the first trimester of pregnancy with serious consequences to the fetus. Despite increased episodes of rubella epidemics (127 outbreaks in 2009–2015), rubella national vaccination is not yet introduced in Ethiopia. In January 2018, an increase of fever and rash cases was reported in Kuyu District of Oromia. We investigated the outbreak to confirm rubella, determine risk factors, and guide interventions.

**Methods:**

We identified rubella cases from health centers and conducted a case-control study (1 case : 2 controls) with 150 participants, from March 12 to 15, 2018. Cases were people who presented with fever and rash or laboratory-confirmed cases. Controls were age matched (<15 yrs) with neighbors selected purposively. We interviewed parents by a structured questionnaire and observed the housing condition. Variables include sex, age, vaccination status, family size, contact history, housing condition, and travel history. Simple logistic regression was used to select the candidate variable at a *P* value <0.25. We identified risk factors at *P* < 0.05 with AOR and 95% CI by multivariate logistic regression.

**Results:**

We identified 50 cases (with no death), and out of them, seven (14%) were confirmed cases (rubella IgM positive). The mean age of the cases was 6 ± 3 years and of the controls was 8 ± 4 years. Family size >5 (AOR = 2.4; 95% CI: 1.5–4.11), not well-ventilated living room (AOR = 4.7; 95% CI: 3.43–8.12), history of contact with rash people (AOR = 2.2; 95% CI: 1.6 3.5), no history of diarrhea in the last 14 days (AOR = 0.8; 95% CI: 0.6–0.9), and no history of vitamin A supplementation (AOR = 2.9; 95% CI: 1.7–2.6) were significant factors for rubella infection.

**Conclusions:**

We identified rubella outbreak in the rural area. Crowded living condition, large family size, not receiving vitamin A in the last 6 months, and contact with people with symptoms of rubella were factors that drove the outbreak, while not having diarrhea in the last 14 days was the protective factor. We recommended the introduction of rubella immunization national programs and advocated the policy on rubella vaccine and strengthening surveillance for congenital rubella syndrome and rubella.

## 1. Background

Rubella is a vaccine-preventable disease that mainly goes unnoticed leading to significant morbidities. Infection in early pregnancy results in serious consequences to the fetus. It is an infectious disease caused by an enveloped RNA virus of the Togaviridae family and the *Rubivirus* genus, for which humans are the only known host [1].

Rubella virus is transmitted by the respiratory route, and initially, it replicates in the nasopharyngeal mucosa and local lymph nodes. Postauricular lymphadenopathy, occipital lymphadenopathy, and posterior cervical lymphadenopathy are its characteristics, and they typically precede the rash by 5–10 days. The maculopapular, erythematous, and often pruritic rash occurs in 50–80% of rubella-infected persons. The rash, usually lasting 1–3 days, starts on the face and neck before progressing down the body. Its incubation period ranges from 12 to 23 days, with an average of 14 days [2–4].

With the goal of extending the full benefits of immunization to all persons, the Global Vaccine Action Plan (GVAP) 2011–2020 was outlined and endorsed by the World Health Assembly in 2012. Towards achieving GVAP goals, rubella vaccine had been introduced in 149 of 194 WHO member countries as of September 2016 [5, 6].

Although rubella is a vaccine-preventable disease, rubella vaccination is not part of the Expanded Program on Immunization in Ethiopia. As part of rash surveillance, clinicians across the country are requested to send blood samples from persons meeting the measles case definition to the national laboratory for measles serology testing, where they are also tested for rubella antibodies. Currently, Ethiopia has planned to introduce measles-rubella vaccine into the routine Expanded Program on Immunization schedule for children under 1 year of age in 2019 [7, 8].

Rubella infection is endemic, and it circulates widely in Ethiopia. In the years 2009–2015, about 127 rubella virus outbreaks were identified by laboratory confirmation in Ethiopia. The number of outbreaks increased dramatically from 3 in 2009 to 38 in 2013 and dropped to 23 in 2015. The highest rubella IgM positivity rate (20.7%) and the highest number of positive cases (1,103) were found among children aged 5–9 years [9]. A majority of the laboratory-confirmed cases were detected from the central and western parts of Ethiopia, and the highest was in Oromia Region (29.3% of all confirmed cases) [7, 8].

On January 28, 2018, North Zone Health Department received report of four suspected cases of measles with symptom of rash and fever from Kuyu General Hospital. The investigation team comprising an epidemiologist, a Public Health Emergency Management (PHEM) surveillance officer, a medical doctor, laboratory personnel, a environmental health professional, and a pharmacist were mobilized to the field on February 5, 2018, to get more information to confirm the outbreak and rule out other possible diseases. The team collected more information about demographics and clinical findings from 50 suspected cases, and ten serum samples were collected. The team collected data from all the suspected cases using the Line Listing tool. A majority (34; 85%) of the suspected cases were present with rash and fever. The serum samples collected were sent to National Laboratory Center in Addis Ababa, Ethiopia.

Results of the laboratory test were released after 7 days. Among ten serum samples, seven were positive for the rubella IgM test, but none of them were positive for the measles IgM test. Based on the suggestive clinical findings and laboratory test results, the condition was confirmed and declared as rubella outbreak in the district. However, little is known about the context under which rubella infection was acquired in the Ethiopian setup. This could be due to the poor attention given to the disease condition by researchers, caregivers, Ministry of Health, and other stakeholders in the country. Therefore, the aim of this investigation was to determine factors driving transmission of rubella infection in children in Kuyu District, Ethiopia.

## 2. Methods

### 2.1. Study Setting

**Figure 1:**
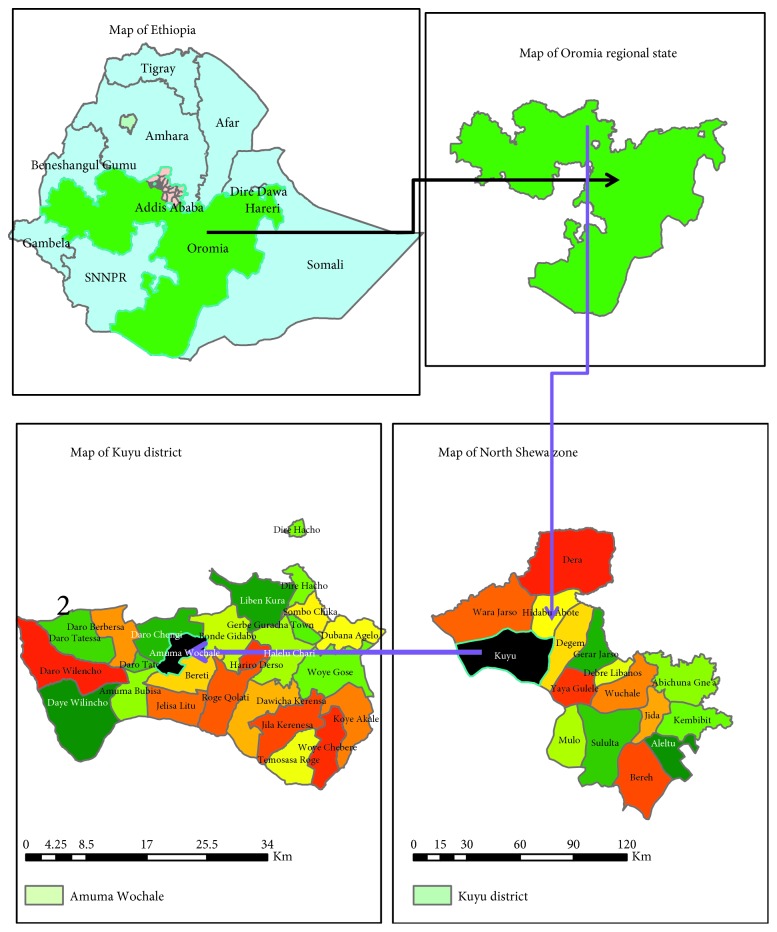
Map of Amuma Wuchale Kebele, Kuyu District, North Shewa Zone, Oromia Region, 2018.

This investigation was conducted in Kuyu District which is found 156 kilometers away from Addis Ababa to the northern part of Oromia Region (Figure 1). Administratively, the district has twenty-three rural kebeles (the smallest administrative unit in Ethiopia) and three towns. According to population projection of 2007, the total population of the district in 2018 was estimated to be 152,556, of which 75,924 were males. According to the population projection, there were 5,157 children under 1 year, 23,951 under 1–5 years, and 68,650 under 5–14 years. Cases of rubella outbreak were found in only one kebele in Kuyu District, Amuma Wuchale. The kebele had a total of 3,201 people, of which 593 were children under five years. It has one functional health post and one 1^st^ cycle school with a total of 674 students. Data were collected from January 16, 2018, to March 18, 2018.

### 2.2. Study Design

A community-based case-control study was employed.

### 2.3. Population

#### 2.3.1. Cases

Cases are any person less than 15 years who is residing in Amuma Wuchale Kebele of Kuyu District with laboratory-confirmed rubella or who meets clinical definition (fever with rash) and is epidemiologically linked to a confirmed case of rubella between January 16, 2018, and March 18, 2018.

#### 2.3.2. Controls

Controls are any person less than 15 years of age who resides in the same village with the cases that did not have any clinical manifestation (signs and symptoms) of rubella between January 16, 2018, and March 12, 2018.

### 2.4. Sampling

There were 50 identified cases of rubella, and 49 of them were included in the study as cases and one case was excluded because of age (34 years). We considered 100 controls based on the ratio of 1 case : 2 controls (very small sample). Controls were selected by the nonprobability sampling technique starting with the household closest to that of the case patient, and subsequent households were visited until an eligible control was found and enrolled. A control living in a household closest to the case's household was selected.

### 2.5. Data Collection

#### 2.5.1. Active Case Searching

There were 246 households in the affected village, Amuma Wuchale Kebele, and all 246 households were visited for active case search by talking to parents. All children less than 15 years were screened for rubella disease using case definition. We also visited Wuchale Primary School and screened all the children attending the school. Line Listing was used during reporting of suspected rubella cases. Data collected by both active and passive surveillance using the data collection tool and Line Listing at Brity Health Center were used. Clinical definition (suspected case of rubella) includes manifestations like fever, cough, runny nose, conjunctivitis, and generalized body rash. All children less than 15 years were screened based on these manifestations.

A structured questionnaire adopted from the updated WHO measles and rubella outbreak investigation guideline [2, 4] was used to collect data from cases and controls. Data about sociodemographic variables, vaccination status, vitamin A supplementation status, contact history, and household factors were collected by nurses working in the nearby health center. One day orientation was given to data collectors. Completeness of data was verified by a principal investigator on daily bases. Data were coded, organized, and entered into EpiData software version 3.1 by double entry for verification.

#### 2.5.2. Document Review

We reviewed medical records of patients at Brity Health Center and program report on the Expanded Program on Immunization at the district level.

#### 2.5.3. Blood Sample Collection

The serum sample of suspected cases of rubella was collected and linked with a case-based reporting form showing patient identification information. The serum sample and identification information were collected by a laboratory technician working at Brity Health Center. From each ten suspected cases, 5 ml of blood was collected by venipuncture into a duly labeled sterile tube. The samples were transported to a central special laboratory, the Laboratory of Ethiopian Public Health Institute in Addis Ababa, Ethiopia, for confirmation of a specific pathogen. During transporting, the standard protocol was maintained for shipping of the serum sample to the central laboratory.

### 2.6. Data Analysis

Collected data were entered into the EpiData software. Data were exported to and analyzed by the SPSS version 20 statistical software. Descriptive statistics were done to generate summary measures (proportion, mean, and standard deviation). The outcome variable was rubella infection. Factors driving rubella infection transmission were determined using the logistic regression model. Variables significant at a *P* value less than 0.25 on bivariate logistic regression were considered candidate variables for multivariate logistic regression. Adjusted odds ratio (OR) with its 95% confidence level (95% CI) was reported.

## 3. Results

### 3.1. Outbreak Description

A total of 50 rubella cases with no death were seen between January 19, 2018, and February 10, 2018, at Amuma Wuchale Kebele, Kuyu District, Oromia Region, Ethiopia. Seven cases were laboratory confirmed, and the rest were epidemiologically linked cases. Of these, 27 (55.1%) were females, showing a female-to-male ratio of 1.2 : 1. Twenty-one (40.8%) cases were under five years of age; twenty-eight (55.1%) cases were children of 5–14 years, and 1 (4.1%) was a woman of child-bearing age (15 to 49 years) with the age ranging from 1 to 34 years. The mean age of the cases was 7 (SD ±5.54) years. The rubella attack rate was 1.7% in females and 1.4% in males in the district. Children less than five years were highly affected with the rubella attack rate of 3.5%, and the rate was 3% among children of 5–14 years (Table 1).

**Figure 2:**
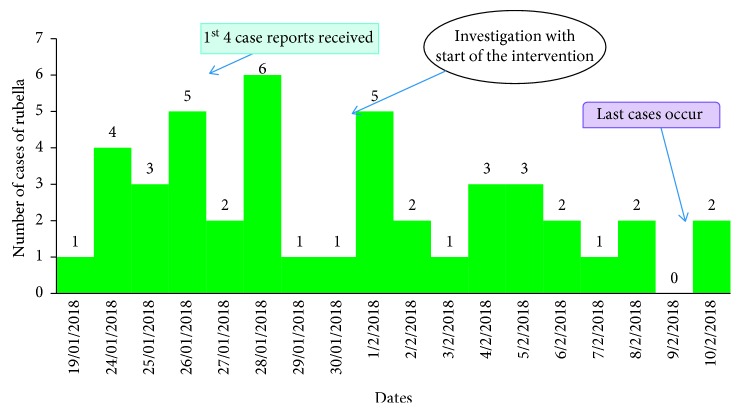
Epidemic curve of rubella cases on outbreak in Amuma Wuchale Kebele, Kuyu District, North Shewa Zone, Oromia Region, February 2018.

Twenty-seven (55.1%) cases were children from Wuchale Primary School, and the other cases were children who do not attend school. The peak of the outbreak occurred on January 28, 2018, while 60% of all cases occurred in the first 8 days of the outbreak. This was followed by a gradual decline but with several smaller intermittent peaks. The last case occurred on February 10, 2018. The epidemic curve shows several peaks suggesting a propagated outbreak. There was a sharp increase of cases to a peak of 35 cases on February 1, 2018. There was a gradual decrease in the number of cases from February 5 until February 11 where they were completely controlled (Figure 2).

### 3.2. Risk Factors for Rubella Infection

A total of 49 cases and 100 controls of children below 15 years were recruited into the study. Of the 49 cases, 22 (44.9%) were males. Of the 100 controls, 55% were males and the rest were females. The mean age of the cases was 6 ± 3 years and of the controls was 8 ± 4 years (Table 2).

Among the total cases, 36 (73.46%) were vaccinated for measles, while 86 (86%) controls were vaccinated for measles, and 55 (64%) from all participants had their vaccination cards. About 83% of cases and 88% of controls were vaccinated for measles only once, while the rest had received more than one doses.

On bivariate analysis, statistically significant factors at a *P* value ≤ 0.25 for contracting rubella in Amuma Wuchale of Kuyu District were family size of greater than five children in the house (COR = 1.4; 95% CI: 0.5–2.01), having contact with cases (COR = 2.7; 95% CI: 1.4–3.8), not well-ventilated living room (COR = 2.7; 95% CI: 1.3–5.7), travel history (COR = 4; 95% CI: 0.89–6.2), no knowledge on rubella (COR = 1.65; 95% CI: 0.8–3.4), no history of diarrhea in the last 14 days (COR = 0.4; 95% CI: 0.2–0.9), no history of vitamin A supplementation in the last six months (COR = 4; 95% CI: 1.1–12), and contact history with case patients (COR = 2.7; 95% CI: 1.4–3.8) (Table 3).

#### 3.2.1. Risk Factors for Contracting Rubella

On multivariate logistic regression, factors independently associated with rubella infection were family size of greater than five children in the house (AOR = 2.4; 95% CI: 1.5–4.11), not well-ventilated living room (AOR = 4.7; 95% CI: 3.43–8.12), history of contact with people who had rash, (AOR = 2.2; 95% CI: 1.6–3.5), no history of diarrhea in the last 14 days (AOR = 0.8; 95% CI: 0.6–0.9), and no history of vitamin A supplementation in the last six months (AOR = 2.9; 95% CI: 1.7–2.6) (Table 4).

Children living with more than five family members were 2.4 times more likely to get infected by rubella than those who were with fewer family members. The odds of infection by rubella for children living in not well-ventilated house were 3.4 times higher than those for children living in well-ventilated house. Having contact with people with rash increases the odds of infection by rubella by 2.2 when compared to those who did not have contact history. Not having diarrhea in the last 14 days prevents risk of infection by rubella by 25% as compared to having diarrhea. The odds of infection by rubella for children who did not receive vitamin A in the last six months were 2.9 times higher than those for children who received it.

## 4. Discussion

According to this investigation, large family size, poor household ventilation, contact history with rubella-like infections, prior diarrheal disease, and no supplementation with vitamin A predispose children to rubella infection in Ethiopian districts. The driving factors for rubella infection transmission were related to the household level and nutritional factors. Odds of acquiring rubella infection were higher among children living in poor-ventilated rooms. This was consistent with the result of the study done in Zimbabwe, Douglas County in Latin America, Tanzania, and Rural Kenya, where poor-ventilated living room of the household was a significant risk factor for contracting rubella [10–13]. This finding is biologically plausible considering that rubella spreads through respiratory secretions.

The odds of acquiring rubella infection among children from households with family size greater than five were 2.4 times higher than those among children with family size less than five. This was consistent with a study of rubella outbreak investigation done in India, Zimbabwe, and Metekel Zone of Ethiopia whereby overcrowding at home was a risk factor for contracting rubella [12, 14]. This implies that having more children in the household increased the risk of being in contact with the infected child.

We found that children with contact history with people with symptoms had 2.2 times higher odds of rubella infection. This finding is consistent with the finding of outbreak investigation in Metekel Zone, Binishangul Gumuz Region of Ethiopia [14]. In addition to this, during active case search, we identified that the index case for each affected household was children from Wuchale Primary School. However, children continued to attend the school and infected children were not isolated at the family level, thereby spreading the disease to other children.

Not having diarrhea in the last 2 weeks had 25% reduction in risk of acquiring rubella infection compared with having diarrhea. Not suffering from diarrhea 2 weeks before was a protective factor for contracting rubella; this is consistent with the results of the study in Kenya where no history of diarrhea was a protective factor [15]. This result is consistent with diarrhea predisposition under five-year children for many infectious diseases including rash illness [3, 5]. This might be explained by the effect of diarrhea in reducing immunity of young children, hence predisposing them to common infectious diseases.

The odds of acquiring rubella infection among children with no history of vitamin A supplementation in the last six months were 2.9 times higher than those among children who had been supplemented with vitamin A. This is consistent with the finding of rubella outbreak investigation conducted in Poland where not taking vitamin A in the last month had 1.9 times higher odds of acquiring rubella infection [16]. This can be explained by the physiological effect of vitamin A on prevention of rash diseases.

### 4.1. Limitation

Ascertainment bias might be introduced because there is a possibility that controls could have been infected with rubella virus but not yet developed signs and symptoms of rubella during the investigation period, which might have introduced misclassification bias. Recall bias for variables like diarrheal disease, contact history with cases, and vitamin A supplementation history could occur. Caregivers or respondents might not take any mild diarrheal disease seriously and could respond no history diarrheal disease. The small sample of cases could also affect the power of the study and might be unable to detect true association. This study was conducted on children who were less than 15 years; hence, the predisposing factors identified might not be the risk factors in adults. Therefore, readers of this paper should take into account these possible limitations when using the findings.

## 5. Conclusion

Rubella outbreak was driven by contact at school and was spread into the community through school children. Not taking vitamin A in the last 6 months, large family size, and poor ventilation of living rooms were the major factors pooling the outbreak, while not having diarrhea in the last fourteen days was a protective factor for rubella infection.

### 5.1. Public Health Actions Done

The following activities were done in order to control the rubella outbreak: sensitization of health workers, village health workers, school teachers, school children, and the community at large; active case finding in schools and in the community; isolation of cases both at school and in the community; providing measles vaccine to all unvaccinated children in the village; and giving health education about rubella both at school and to community members. Feedback was given to the district.

### 5.2. Recommendation

District health office should work towards improving housing condition, regular provision of vitamin A, strengthening surveillance for congenital rubella syndrome especially in children under one year and pregnant mothers, and strengthening family health program.

Ministry of Health should develop rubella outbreak investigation and response guideline and introduce rubella containing measles vaccine within the national immunization program by adopting the WHO policy on rubella vaccination which currently recommends combining measles and rubella control strategies and planning efforts which focus on the widespread use of measles-rubella vaccine. Furthermore, a large study is needed for exploration of other variables.

## Figures and Tables

**Table 1:** Sex- and age-specific attack rate of rubella cases in Amuma Wuchale Kebele, Kuyu District, North Shewa Zone, Oromia Region, 2018.

Variable		Population	Number of cases	Attack rate (%)
Sex	Male	1600	23	1.4
	Female	1550	27	1.7

Age	0–4 years	593	21	3.5
	5–14 years	906	28	3
	≥15 years	1702	1	0.12

**Table 2:** Sociodemographic characteristics of cases, controls, and caregivers in Amuma Wuchale Kebele, Kuyu District, North Shewa Zone, Oromia Region, 2018.

Variables	Categories	Number of cases (%)	Number of controls (%)
Age	≤5 years	21 (40.8)	30 (30)
	5–14 years	28 (55.1)	70 (70)

Sex	Male	22 (44.9)	55 (55)
	Female	27 (55.1)	45 (45)

Religion	Orthodox	48 (96)	93 (93)
	Others	2	7

Ethnicity	Oromo	47 (95.9)	90 (90)
	Others	3 (4.1)	10 (10)

Occupation	Farmer	33 (67.4)	68 (68)
	Housewife	6 (12.2)	13 (13)
	Government worker	10 (20.4)	19 (19)

Parents' educational status	Illiterate	6 (12.2)	13 (13)
	Primary	19 (38.8)	45 (45)
	Secondary and above	24 (49)	42 (42)

Family size	<5	18 (36.8)	44 (44)
	>5	31 (63.2)	56 (56)

**Table 3:** Factors associated with rubella infection on bivariate analysis in Amuma Wuchale Kebele, Kuyu District, North Shewa Zone, Oromia Region, 2018.

Variables	Categories	Number of cases (%)	Number of controls (%)	COR (95% CI)	*P* value
Family size	≤5	3 (6.1)	13 (13)	1	0.14
	>5	46 (93.9)	87 (87)	1.4 (0.5–2.01)	

Contact history	Yes	33 (67.3)	24 (24)	2.7 (1.4–3.8)	0.042
	No	16 (32.7)	76 (76)	1	

Ventilation of rooms	Yes	26 (53)	31 (31)	1	0.006
	No	23 (47)	69 (69)	2.7 (1.3–5.7)	

Travel history	Yes	4 (8)	4 (4)	4 (0.89–6.2)	0.205
	No	45 (92)	96 (96)	1	

History of diarrhea	Yes	33 (67.35)	45 (45)	1	0.031
	No	16 (32.65)	55 (55)	0.4 (0.2–0.9)	

Knowledge on rubella	Yes	29 (59.2)	50 (55)	1	0.18
	No	20 (40.8)	40 (45)	1.65 (0.8–3.4)	

Taking at least one dose of measles vaccine	Yes	36 (73.5)	86 (86)	0.84 (0.25–0.93)	0.038
	No	13 (26.5)	14 (14)	1	

History of VA supplementation	Yes	12 (24.5)	9 (9)	1	0.009
	No	37 (75.5)	91 (91)	4 (1.1–12)	

COR, crude odds ratio; CI, confidence interval; VA, vitamin A.

**Table 4:** Independent factors for contracting rubella in Amuma Wuchale Kebele, Kuyu District, North Shewa Zone, Oromia Region, 2018.

Variables	Categories	COR (95% CI)	AOR (95% CI)	*P* value
Family size	≤5	1	1	0.046
	>5	1.4 (0.5–2.01)	2.4 (1.5–4.11)	

Ventilation of rooms	Yes	1	1	0.0001
	No	2.7 (1.3–5.7)	4.7 (3.43–8.12)	

History of contact	Yes	2.7 (1.4–3.8)	2.2 (1.6–3.5)	0.032
	No	1	1	

History of diarrhea	Yes	1	1	0.012
	No	0.4 (0.2–0.7)	0.8 (0.6–0.9)	

History of VA supplementation	Yes	1	1	0.025
	No	4 (1.1–12)	2.9 (1.7–2.6)	

COR, crude odds ratio; AOR, adjusted odds ratio; CI, confidence interval; VA, vitamin A.

## Data Availability

Data that support the finding of this study are available from the corresponding author, but restriction applies to the availability of these data, which were used under license for the current study and so are not publicly available.

## Abbreviations

AFRO: African Regional Office
CDC: Center for Disease Control
CRS: Congenital rubella syndrome
EFETP: Ethiopian Field Epidemiology Training Program
EPI: Expanded Program on Immunization
IgM: Immunoglobulin M
MCV: Measles-containing vaccine
MMR: Measles, mumps, and rubella
PHEM: Public Health Emergency Management
SIA: Supplemental immunization activity
UNICEF: United Nations International Children's Emergency Fund
WHO: World Health Organization.
